# A Case of Remarkable Vitality

**Published:** 1904-09-24

**Authors:** 


### A CASE OF REMARKABLE VITALITY.

An example of extraordinary human endurance is published in the *Guy's Hospital Gazette* of August 27th. The patient, a boy of 14 years, was admitted to hospital with an abscess in the right iliac region. A few hours after admission, on April 22nd, he was anæsthetised; there was some struggling during the preliminary stages of anæsthesia and the iliac swelling became much less marked. On the abdomen being opened in the region of the appendix some foul pus escaped, though not so much as had been expected. Signs of general peritonitis rapidly became manifest and on the following day the abdomen was incised near the middle line; a large quantity of pus escaped and the peritoneal cavity was thoroughly washed out and drained. On April 27th a subdiaphragmatic abscess was suspected on the right side, and an aspirating needle being inserted in the ninth intercostal space behind foul *Bacillus coli* pus was withdrawn. Part of the ninth rib was therefore resected, the diaphragm was sewn to the parietal pleura and a quantity of pus was evacuated from beneath the diaphragm. Cellulitis over the right side of the thorax developed and on April 29th, under an anæsthetic, six incisions from two to four inches long were made in the cellular tissues giving vent to a quantity of thick pus. On April 30th the cellulitis had increased, and the affected area was therefore incised again and scraped. At the end of three days a fæcal fistula developed in the second abdominal wound, and, constantly discharging, caused severe excoriation of the skin of the abdomen, and produced great emaciation from malnutrition. Subsequently a subdiaphragmatic abscess developed on the left side and by aspirating through the eighth left intercostal space pus was withdrawn. On May 11th the eighth left rib was resected and the abscess cavity drained. On May 24th an operation was performed with a view to closing the fæcal fistula. Later an empyema was discovered in the right chest, and on June 7th resection of part of the eighth rib was carried out, and the empyema drained. After this last operation the patient made an uninterrupted recovery. It is remarked that "throughout his illness the boy remained cheerful, hopeful, and very matter of fact, consenting to any operation without complaint, and even taking an interest in what was going to be done to him"—a disposition that must in great measure have assisted towards restoration of health.

Though the patient's tenacity of life in this instance was exceptional, the case serves to point a moral, namely, that *Bacillus coli* abscesses containing foul-smelling pus as a rule produce slight constitutional effects in comparison with acute abscesses due to other infective agents, and that so soon as a vent is given to the fœtid pus the constitutional symptoms are often very rapidly relieved, facts which have an important bearing both on the prognosis and treatment of these conditions.

